# Evaluation of a cross-sectoral care intervention for families with psychosocial burden: a study protocol of a controlled trial

**DOI:** 10.1186/s12913-022-07787-9

**Published:** 2022-04-11

**Authors:** Gloria Metzner, Sabine Horstmann, Michael Barth, Jürgen M. Giesler, Susanne Jünemann, Klaus Kaier, Christian Schlett, Nora Schroeder, Marcus Siebolds, Frank Sinss, Juliane van Staa, Manuela Glattacker, Ilona Renner

**Affiliations:** 1 Section of Health Care Research and Rehabilitation Research, Institute of Medical Biometry and Statistics, Faculty of Medicine and Medical Center, University of Freiburg, Hugstetter Str. 49, 79106 Freiburg, Germany; 2 National Centre for Early Prevention, Federal Centre for Health Education, Maarweg 149–161, 50825 Köln, Germany; 3 Center for Pediatrics, Medical Center, University of Freiburg, Mathildenstraße 1, 79106 Freiburg, Germany; 4 Institute of Medical Biometry and Statistics, Faculty of Medicine and Medical Center, University of Freiburg, Zinkmattenstr. 6a, 79108 Freiburg, Germany; 5 Department of Health Care, Catholic University of Applied Sciences, Wörthstraße 10, 50668 Köln, Germany

**Keywords:** Family, Small children, Psychosocial burden, Pediatricians, Prevention, Early childhood intervention program, Supportive services, Complex intervention, Cross-sectoral care, Evaluation

## Abstract

**Background:**

Family risk factors, e.g. low socioeconomic status or parental mental health disorders, can affect children’s health and development. Thus, targeted preventive services for families with psychosocial burden are crucial. The German Early Childhood Intervention (ECI) program is a preventive approach that aims to strengthen parent’s resources by supportive services. However, research has revealed that only a proportion of the families considered to have substantial risk factors access the ECI program. To increase pediatricians’ skills in identifying risk factors, and to improve the cross-sectoral collaboration between relevant professionals and the referral of families to supportive services, the PATH-intervention (Pediatric Attention To Help) was developed. The PATH-intervention includes interprofessional quality circles and a one-day training program for the pediatricians. This study aims to evaluate this complex cross-sectoral care intervention for families with psychosocial burden.

**Methods:**

Using a prospective quasi-experimental, controlled (matched-pair), longitudinal mixed-method design, we will compare families under treatment of pediatricians trained in the PATH-intervention with families under treatment of a control group of pediatricians. Participating families are asked to complete online-surveys. As a primary outcome, we will examine the use of supportive services of the ECI by burdened families. Secondary outcomes are the proportion of correctly identified families with psychosocial burden by the pediatricians, as well as information provision and motivation of the families to use the supportive services. Additionally, the cost-effectiveness ratio will be investigated. In the process evaluation, we will qualitatively explore the acceptance of the PATH-intervention of all involved stakeholders and the treatment fidelity of the trained pediatricians.

**Discussion:**

This study will determine whether the PATH-intervention enables the pediatricians to identify and recommend supportive services to burdened families, as well as the families’ use of the supportive services of the ECI. Qualitative data will give insight into the acceptance of the intervention from the perspective of all stakeholders and the treatment fidelity. Results of this study could be the starting point for the broader implementation of the PATH-intervention as standard care.

**Trial registration:**

German Clinical Trials Register (DRKS): DRKS00023461 (3rd December 2020); WHO UTN: U1111- 260-6575.

**Supplementary Information:**

The online version contains supplementary material available at 10.1186/s12913-022-07787-9.

## Background

In the last two decades, increasing attention has been paid to risk factors affecting children’s health and development [1–3]. Risk factors include, among others, low socioeconomic status, parental mental health disorders (e.g. depression), parental adverse childhood experiences, unplanned pregnancy or early parenthood, domestic violence and substance abuse [1, 4, 5]. There is evidence that an accumulation of risk factors affects children’s wellbeing, health and developmental outcomes, and can also influence physical and mental health in later adult life [1, 6].

In Germany, a representative survey of families with small children (aged 0 to 3 years) revealed that 12.9% of the families reported four or more risk factors [5], indicating a high psychosocial burden on those families. Similar results were found in a representative survey among pediatricians: 13.8% of the families visiting a preventive medical check-up for their child were considered to have notable risk factors. More precisely, pediatricians rated these families as being under psychosocial stress, which could have a significant impact on the further development of the children involved. Frequently reported issues were being a single parent, low educational level, poverty, and parent exhaustion [7].

Given the negative consequences of risk factors on children’s development, targeted preventive services for families with psychosocial burden and children at risk are crucial. The German Early Childhood Intervention (ECI) program (“Frühe Hilfen”) represents a nationwide implemented preventive approach that aims to strengthen parent’s resources through supportive services. For example, these voluntary services comprise long-term home visiting services by healthcare professionals who have additional qualifications in psychosocial care (family midwifes or nurses), specific counseling services, or parent-child-groups. Such services can have benefits for the further development of children and, from a socioeconomic perspective, society as a whole [8, 9].

In order to overcome the “prevention dilemma”, which describes that especially families with particularly high needs do not use supportive services [10], improved cross-sectoral collaboration between professionals of health and social services is needed. In this context, pediatricians play an important role. Commonly, families with small children regularly visit pediatricians for medical check-ups – the so-called “U-Untersuchungen”. These medical check-ups for infants span from birth until the age of five (named U1 to U9). Over 95% of the families with small children make use of these preventive check-ups – throughout all social classes [11]. Thus, these check-ups provide a good opportunity to detect risk factors of families and to recommend regional supportive services.

However, research revealed that only one in every six families that are considered by a pediatrician to have substantial risk factors, actually access the ECI program [7]. One possible reason for this situation might be that the pediatricians frequently adopt a wait-and-see position. Furthermore, pediatricians and parents predominantly address psychosocial burden indirectly and rarely discuss them in depth [12]. Lastly, one in every five pediatricians find it difficult to identify psychosocial burden and risk factors during a medical check-up [7]. They also report a lack of time and inappropriate financial compensation for counseling.

To increase pediatricians’ skills to identify risk factors and their conversation skills to discuss psychosocial burden with families, as well as to improve the cross-sectoral collaboration between relevant professionals and the referral of families to the supportive services of the ECI, the National Center for Early Prevention (NZFH) developed the PATH-intervention (Pediatric Attention To Help) in cooperation with the Association of Statutory Health Insurance Physicians of the German state of Baden-Württemberg [13].

So far, no comprehensive evaluation of this complex healthcare intervention has been conducted. Considering the potential benefits of the ECI services on the further development of children, the overall objective of this study is to evaluate the PATH-intervention, which aims to facilitate a precise referral of families with small children (aged 0-3 years) and psychosocial burden to the supportive services of the ECI.

## Method

### Intervention

The PATH-intervention aims to promote cross-sectoral collaboration between pediatricians, child and youth welfare services (CHWS), and further agents of healthcare and psychosocial supportive services for burdened families with small children (aged 0-3 years). Its goal is a more frequent and targeted referral of families to the ECI system. The PATH-intervention comprises two central elements: interprofessional quality circles (IQCs) and a one-day training program for pediatricians.

Since 2010, the Association of Statutory Health Insurance Physicians of the German state of Baden-Wuerttemberg established stepwise-accredited interprofessional quality circles that include pediatricians as well as social workers and others (e.g. gynecologists, psychotherapists) from the CHWS and ECI services. Specially trained moderating tandems consisting of pediatricians and staff members from CHWS lead the IQCs [13]. The IQCs take place twice a year. The topics are case conferences on family burden. These case conferences target the planning of preventive measures to support the burdened family from an interprofessional perspective. Other issues of the IQCs are the discussion of developmental risks of children and becoming more familiar with supportive services in the respective regional area.

The one-day training program compiled by M. Siebolds and B. Münzel (Siebolds M, Münzel B: Schulungsunterlagen zur Ausbildung der Moderatorentandems für IQZ sowie für die Instrumente der Fallfindung und des motivierenden Elterngesprächs, unpublished) aims to enhance the pediatricians’ skills to facilitate the family’s disclosure of sensitive information about the child’s care situation, the family’s burden and resources, to explore the family’s motivation of change and to refer them to the local organizations of the ECI, or directly to tailored supportive services. The training focusses on (1) specific aspects of the pediatrician’s interview style, such as questions about psychosocial issues and living conditions, supportive statements and encouragement of parental narration [14]; (2) principles of motivational interviewing (e.g. collaboration, evocation and autonomy supportive) [15]; (3) tailoring interventions to the family’s wish to change [16]; (4) dealing with indications of child maltreatment [17]; and (5) transferring the family to the local organizations of the ECI and supportive services [18]. In cases of uncertainty about the extent of the family’s psychosocial burden, they should use an interview guide to capture specific risk factors (e.g. parental depression, regulation disorders).

### Research aim and hypotheses

In order to evaluate the PATH-intervention, we compare families under treatment of trained pediatricians in the German state of Baden-Württemberg (IG) with those of a control group (CG; pediatricians not participating in the PATH-intervention) in the German state of Bavaria. More precisely, we put forward the following hypotheses: (1) Families with psychosocial burden who are under treatment of an IG-pediatrician use supportive services more often than burdened families treated by a CG-pediatrician (primary outcome). (2) As a precondition for successful referral to supportive services, we assume that pediatricians in the IG are better than their counterparts in the CG at (2a) identifying families with psychosocial burden, (2b) informing them about regional ECI services, and (2c) motivating them to use these services (proximal secondary outcomes). Thus, the proportion of families, who received information and were motivated, will be higher in the IG than in the CG. (3) From a socioeconomic point of view, the ratio between costs and effectiveness of the PATH-intervention will be positive (distal secondary outcome). (4) All involved stakeholders - the families with small children (0-3 years), pediatricians and members of the ECI network - appraise the PATH-intervention in a positive way (distal secondary outcome).

### Study design and setting

**Fig. 1:**
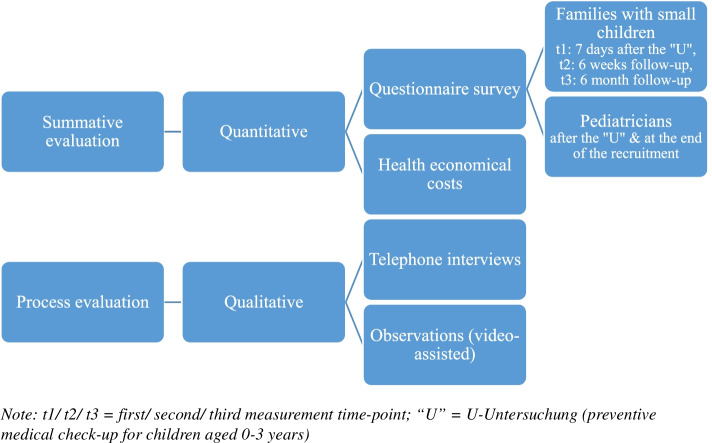
Study design of the PATH study

This study is designed as a prospective quasi-experimental, controlled (matched-pair), longitudinal mixed-method trial [19]. It comprises a summative evaluation, as well as process evaluative study sections (Fig. 1). The summative evaluation is based on quantitative surveys. Participating families with small children (0-3 years) are asked to answer an online-survey at three measurement time-points: up to 7 days after a preventive medical check-up (U3-U7a) at the pediatrician (t1), follow-up at 6 weeks after the doctor’s visit (t2) and again 6 months later (t3). The pediatricians answer a questionnaire immediately after each medical check-up with a participating family. They will also answer a brief questionnaire on confounding factors (e.g. work experience) after the completion of the recruitment of families. Additionally, the evaluation is complemented by health economic analyses, including the analysis of health insurance data. As part of the process evaluation, we will conduct individual telephone interviews with participants of all stakeholder groups involved in the intervention – families, pediatricians and ECI network members. Another part of the qualitative process evaluation are video-assisted observations during the medical check-ups to examine treatment fidelity of the IG-pediatricians.

### Participants and recruitment

Study participants comprise the three groups involved in the intervention process: pediatricians, families with small children (0-3 years), and ECI network members. For all groups, signed informed consent is required for participation in this study.

#### Pediatricians

Pediatricians of the IG have to have passed the intervention training or comparable trainings, and at least two IQCs in the past 2 years. The Association of Statutory Health Insurance Physicians of Baden-Württemberg supports the NZFH in finding eligible pediatricians for participation. Pediatricians of the CG are recruited in collaboration with the Professional Association of Pediatricians of the German state of Bavaria. The NZFH obtains informed consent from all pediatricians willing to participate. Based on a retrospective matched pair approach, CG-pediatricians are matched to IG-pediatricians by gender and regional characteristics of the doctor’s office (rural vs. urban, and socio-economic area). Thus, these potential confounding variables should be homogenously distributed in the two groups. All participating pediatricians get an expense allowance of 60€ per recruited family, and additionally 60€ for participating in the interview study section.

#### Families

Eligible families are all families with small children aged 0 to 3 years undergoing treatment by the participating pediatricians. The pediatricians ask the families for participation during the preventive medical check-ups of the U3 up to the U7a (medical check-ups with the target age of the children) and obtain their informed consent. Language skills in German, Arabic, Italian or Turkish are required for participation. Families, who answer all three surveys receive a 30€ voucher after the last measurement time-point. In addition, families who participate in the interview study section receive an expense allowance of 30€.

#### Members of the ECI network

The members of the ECI networks are municipal network coordinators and stakeholders from the social and health service sector. The participating municipal network members are located in the same regions as the IG-pediatricians and take part in the interview study section. The NZFH recruits the members of the ECI network and an external institute, which will conduct the telephone interviews, will obtain informed consent.

### Procedure

#### Summative evaluation

##### Questionnaire survey

Participating families are asked to complete online-surveys including questions concerning sociodemographic information, psychosocial burden, the use of supportive services, as well as questions about information provision and motivation by the pediatrician. In addition, families are either asked to rate their satisfaction with a specific offer, or to state the reasons why they did not use it (see Table S1, Additional file 1, for the operationalization of psychosocial burden and Additional file 2 for the family questionnaire about supportive services). The survey takes place at three measurement time-points: up to 7 days after the medical check-up (t1), 6 weeks after the medical check-up (t2) and again at 6 months after the medical check-up (t3). The same person should answer all three surveys. At each time-point, participating families receive a link to the online-survey via e-mail. The link is valid for 7 days. The online-survey is available in four languages, namely German, Arabic, Italian and Turkish.

Immediately after a participating family leaves the medical check-up, the pediatrician rates in a short paper-pencil questionnaire whether the family is psychosocially stressed, and if so, the degree of psychosocial burden (Questionnaire for pediatricians, Additional file 3). Moreover, the pediatricians are asked whether they have addressed the perceived psychosocial burden with the family and to rate the family’s need for supportive services. At the end of the recruitment of families, the pediatricians answer a second paper-pencil questionnaire about possible confounding variables, such as the structural characteristics of the doctor’s office and work experience (Descriptive questionnaire for pediatricians, Additional file 4).

#### Process evaluation

##### Interviews

As there are different stakeholders involved in the intervention process, we examine the acceptance of the PATH-intervention in the perspective of the pediatricians, families and ECI network members. Trained interviewers will conduct telephone interviews with participants of all three groups who agreed to this optional study section. We select families for the interviews who are treated by an IG-pediatrician and report psychosocial burden according to the first completed online-survey. To obtain a range of variation in the sample of the families, we employ further selection criteria related to the age and gender of the child. All IG-pediatricians who additionally declared their interest for the interview study section are asked to participate in the telephone interview. The ECI network members will be exclusively recruited for the purpose of the interviews.

##### Observation

For the purpose of the video-assisted observations, we will select a group of participating families by the following criteria: the families are treated by IG-pediatricians, the families report psychosocial burden according to the first completed online-survey, the previous preventive medical check-up was the U3, U4 or U5, and the families agreed to this optional study section. In such cases, the observation of the attending IG-pediatrician takes place during the next preventive medical check-up with the family, the U4, U5 or U6. Afterwards, the researcher on site conducts a short interview with the family. We intend to conduct two observations per participating IG-pediatrician.

### Study measurements and outcomes

Central for answering the research questions is to assess the risk factors and perceived psychosocial burden of the families. We therefore apply an additive index and count up the risk factors asked for, as is commonly used in research and previous studies [1, 20, 21]. The index comprises six domains, namely the (a) family background, (b) parent’s individual preconditions for dealing with care challenges, (c) parent’s psychological health, (d) difficulties during pregnancy and in interaction with the child, (e) special care challenges regarding the child, and (f) problematical caring behavior. Each domain consists of a set of questions asking for specific risk factors such as low educational qualifications, overcrowding, conflicting partnership, depression, early pregnancy or parental strains. In this context, we utilize commonly used instruments, e.g. the Patient Health Questionnaire (PHQ-4 [22, 23]) measuring parents’ depressive symptoms and the German Version of the Parenting Stress Index assessing challenges and the burden of being a parent [24]. In addition, we apply questions previously used in the representative study concerning the psychosocial burden of families with small children in Germany [25]. Table S1 (Additional file 1) shows the domains and assessed risk factors, and their operationalization with references to the applied, published instruments in detail. In the form of a patient-reported outcome, the participating families themselves answer the questions that underlie the assessment of these risk factors in the online-survey. In line with previous studies, which used the same risk factors as indicators for psychosocial burden [20], families meeting three or more risk factors are judged to have considerable psychosocial burdens (for the calculation of the additive index indicating psychosocial burden, please see Additional file 1).

#### Primary outcome

The primary outcome is the use of supportive services by families with psychosocial burden in the course of the study period. The family survey therefore contains a list of 14 predefined supportive ECI services (Table S2, Additional file 5). For each of these services, the families are asked whether they know about it. If this is the case, they are asked whether they have made use of the service or not (yes-no question). These questions were specifically developed for this study (Family questionnaire about supportive services, Additional file 2). Usage is given when burdened families made use of at least one of the 14 supportive services.

#### Secondary outcomes

Secondary outcomes are classified as proximal and distal outcomes. Proximal secondary outcomes are conceptualized as directly affected by the intervention and observable close to the intervention. In contrast, distal secondary outcomes are located more distant to the intervention and are likely influenced by other external factors than the intervention.

One secondary proximal outcome is the proportion of correctly identified families with psychosocial burden by the pediatricians. With one question, the pediatricians are directly asked whether they judge the family as burdened or not (yes-no question; Questionnaire for pediatricians, Additional file 3). Another two secondary proximal outcomes are, on the one hand, the information provision and, on the other, the motivation of the family with psychosocial burden by the pediatrician. To find this out, the list of the predefined supportive services in the family survey includes questions regarding these two aspects (Family questionnaire about supportive services, Additional file 2). Families, who have already stated to know about a specific supportive service, answer whether the pediatrician informed them about it and recommended them to use it.

One secondary distal outcome is the ratio between costs of the intervention and its effectiveness. In a first step, the total costs of the PATH-intervention will be calculated from the healthcare perspective. The following three aspects form part of the costs of the PATH-intervention: (1) the mandatory one-day-training program for the pediatricians, (2) the continuous participation in the IQCs (twice a year), and (3) reimbursement of certain aspects of the PATH intervention (e.g. identification of burdened families, information provision). All of the three aspects are associated with direct costs, which will be collected from the Association of Statutory Health Insurance Physicians of the German state of Baden-Wuerttemberg and outlined from the healthcare perspective. Both the one-day training program and the participation in IQCs are additionally associated with training credit points for the participating pediatricians, which will be monetarized using opportunity costs and outlined as an additional (societal) perspective. As part of a budget-impact analysis, health insurance claim data from the Statutory Health Insurance “AOK” Baden-Württemberg will be inspected in order to identify the number of individuals that might be applicable to the PATH-intervention in the case of a broader rollout of the program.

The second secondary distal outcome is the acceptance of the PATH-intervention in the perspectives of all stakeholders involved in the intervention process. With qualitative telephone interviews, we intend to gain a deeper insight into the perspectives of the trained pediatricians, burdened families and ECI network members. This includes their appraisal of the PATH-intervention as well as perceived facilitating and hindering factors. Semi-structured interview guidelines will be developed individually for all three groups. The interviews will start with a narrative entry followed by specific questions targeting the themes of interest. Themes of the interview guidelines are the collaboration between professionals, the perceived individual benefit of the intervention, the procedure of identification and discussion of psychosocial burden, the paths to the ECI and supportive services, as well as potential barriers for tailored referrals to adequate services. Interview guidelines for ECI network members will focus on interprofessional cooperation and referral of families into the ECI system and they will deliver background information about the specifics of the regional ECI networks. The telephone interviews will last approximately 1 h, will be audio-recorded, and transcribed verbatim.

#### Treatment fidelity

To examine the realization of the PATH-intervention by the IG-pediatricians in their practice, we use video-assisted observations. A video camera is placed in the doctor’s room, thus filming the communication and interaction between the family and the pediatrician. The researcher is not present during the examination. Afterwards, the researcher on site asks the family if the doctor acted in some way different to usual. This short interview serves for detecting the influence of the research situation and the video camera on the doctor’s professional acting.

### Sample size considerations

#### Summative evaluation

In total, we intend to include *N* = 40 pediatricians in the study. In purpose of the planned statistical twins (matched pair approach), each group (IG, CG) should comprise *n* = 20 pediatricians.

Concerning the family sample, we calculated the sample size for the planned analyses (logistic regressions) of the primary outcome (proportion of burdened families making use of supportive services) using the software GPower. The underlying assumptions were a statistical power of 0.80 and a significance level of 5%. Concerning the effect size, we assumed that 50% of the families with psychosocial burden under treatment of IG-pediatricians would use supportive services, compared to only 10% of the burdened families of CG-pediatricians. Furthermore, we assumed that the portion of families with psychosocial burden will be about 15% in the overall sample. About 50% of the eligible families are presumed to participate in the online-survey, from which about 30% will drop out of the study up to the last follow-up measurement time-point. Thus, to detect a significant effect concerning the use of supportive services by families with psychosocial burden contrasting intervention to control group, a total of *N* = 800 families should be asked to participate by the pediatricians, namely *n* = 20 families per each pediatrician.

#### Process evaluation

We intend to interview *N* = 10 pediatricians of the IG and N = 10 ECI network members. Of the participating families, *N* = 20 families under treatment of IG-pediatricians should be interviewed. For these cases, we use purposeful sampling aiming at maximum variability regarding age and gender of the child, as well as concerning the reported psychosocial burdens in the first online-survey.

A similar purposeful sampling is used to select the sample for the observation study section. The observations are conducted with *N* = 14 families of IG-pediatricians. The families are selected based on the following criteria: the family, which is under treatment of the IG-pediatrician, attended the U3, U4 or U5 at the time of recruitment. In addition, the family reports considerable psychosocial burden in the first online-survey.

### Data analysis

#### Summative evaluation

Before hypotheses are tested, we will perform dropout analyses to ascertain the amount of missing values, the mechanism of missingness and imbalances between the groups regarding study dropout and missing values. As families are treated by pediatricians, their data is not independent. Consequently, we will calculate the design effect to examine whether multilevel models should be used [26] that appear appropriate. Hypothesis are thus examined by generalized linear mixed models, in which families’ belongingness to pediatricians are accounted for by a random effect (random intercept) on Level 2. Level 1 comprises the fixed effects, with which the hypotheses are tested. The primary hypothesis will thus be tested by a multilevel binary logistic regression analysis, in which the dependent primary outcome (use of supportive services by burdened families: yes / no) is predicted by the group-variable (IG or CG) as a fixed effect on Level 1. Additionally, propensity score adjustment [27] will be applied to control for imbalances between IG and CG regarding confounding variables of pediatricians and families. The following model represents the main analysis to test our primary hypothesis:

$$\mathrm{Level}\;1:\mathrm{Outcome}=\beta_{0j}+\beta_1\ast\mathrm{group}+\beta_2\ast\mathrm{propensity}\;\mathrm{score}+r_{ij}$$

$$\mathrm{Level}\ 2:{\beta}_{0j}={\gamma}_{00}+{\mathrm{u}}_{0j}$$

In sensitivity analyses (SA) we will perform the main analysis without propensity-score adjustment (first SA) and as complete cases analysis (second SA). In order to consider the time taken for families to use supportive services, we will perform a time-to-event analysis that predicts the cumulative incidence of the use of supportive services by families over the three measurement points (third SA). Furthermore, we will examine if families in the IG use more supportive services than families in the CG. This explorative analysis compares the number of supportive services used by families in the IG and CG. The hypothesis on secondary outcomes will be examined analogous to the main analysis, by means of multilevel binary logistic regression analyses with propensity score adjustment.

#### Process evaluation

##### Cost-effectiveness analysis

The cost-effectiveness of the PATH-intervention will be analyzed by calculating incremental cost-effectiveness ratios (ICERs) for the following primary and secondary outcomes:

1. Additional costs per use of supportive services by families with psychosocial burden.
2. Additional costs per correctly identified family with psychosocial burden by the pediatrician.
3. Additional costs per additional informing of an identified family by the pediatrician about regional supportive services and motivating them to use these services.

All ICERs will be determined separately from the healthcare and societal perspective and 95% confidence intervals will be calculated based on Fieller’s theorem [28].

##### Analysis of the interviews

The transcribed interviews of families are analyzed using the method of content structuring according to the qualitative content analysis by Kuckartz [29]. For this purpose, all data material is categorized and coded.

Prior studies [7, 10] already provide evidence in the areas of interprofessional collaboration, referral of families to the ECI network and utilization of supportive services. On this basis, and framed by the interview guidelines, an initial coding system will be deductively developed in advance. Based on this initial coding system and themes emerging from the interviews, the categories will be inductively differentiated and specified. Two researchers will carry out these analyses and the intercoder consistency will be evaluated.

Interviews of IG pediatricians and ECI network members will be analyzed by a category-based approach or, depending on data material, with other appropriate methods (e.g., summarized descriptions, case-by-case analysis).

With the content structuring of the material available, category-based, topic-oriented evaluations can be carried out in the further analysis, i.e. how often, or for how many persons, a content-related category (or also a category combination) was coded, and how categories/topics are related. The presentation of results can then be based on summary descriptions supported by meaningful quotations.

##### Analysis of the observation

Treatment fidelity of the complex intervention will be analyzed in a three-step procedure, which will be conducted independently by two researchers and subsequently brought together. First, we document the topics discussed concerning the family’s burden, psychosocial issues, living conditions and the family’s resources, as well as in regard to supportive services. For that, we use a systematically developed schedule, which comprises aspects of the PATH-intervention. For example, the schedule includes aspects of the interview guide, which pediatricians should use in the case of uncertainty about the extent of the family’s psychosocial burden. We record the discussed topics and complement this with the information of the researchers’ observations such as facial expressions and gesture. Additionally, the table includes whether the discussed topic is marked as concern or distress, either by the pediatrician or by parents.

Secondly, we focus on the pediatrician’s conversation style and prevention-promoting attitude, which are core elements of the PATH-intervention. Thus, we rate the conduction of the parental conversation and consultation by using the two central dimensions of Motivational Interviewing, *partnership* and *empathy* [30]. Partnership describes the extent of participatory involvement of parents and the shared expert roles of the pediatrician on the one hand, and parents on the other [30]. Empathy represents the pediatrician’s efforts to understand the parental perspective and to reflect this understanding to the parents [30].

The third step is a conversation analysis of the documented conversation passages of step 1. This analysis focusses on the way the topics are discussed e.g. active listening, appreciation, repeating and summarizing the parents’ contributions. In this step, we categorize different types of questions [31] used by the pediatrician in terms of the extent of evocation of parental perspectives. We expect that pediatricians of the intervention group prefer using open questions and narrative (follow-up) questions for this purpose, based on the skills learned in the one-day training program of the PATH-intervention [32]. Furthermore, a balance index [33] will help to evaluate how often the contributions of the pediatrician refer appropriately to the parents’ contribution and vice versa.

Table 1 displays a summary of the outcomes, measurements, data source and measurement time-points, as well as methods of analysis.

**Table 1:** Summary of outcomes, measurements, data source and measurement time-points, and methods of analysis

Outcomes	Measurement	Data source	Measurement time-point			Data analysis
			t1	t1	t2	
Use of supportive services by families with psychosocial burden (primary outcome)	Online-survey	Families	x	x	x	Comparison of intervention & control group using generalized linear mixed models
Correctly identified families with psychosocial burden (secondary outcome, proximal)	Paper-pencil-questionnaire	Pediatricians	x			
Information provision & motivation of families with psychosocial burden by the pediatrician (secondary outcome, proximal)	Online-survey	Families	x	x	x	
Cost-effectiveness ration of the PATH-intervention (secondary outcome, distal)	Direct costs of the intervention, health insurance claim data	Association of Statutory Health Insurance Physicians Baden-Wuerttemberg, Statutory Health Insurance “AOK” Baden-Württemberg	Throughout the study.			Calculation of incremental cost-effectiveness ratios (ICERs)
Acceptance of the PATH-intervention (secondary outcome, distal)	Telephone interviews	Families with psychosocial burden, IG-pediatricians, ECI network members	Throughout the online-survey data collection phase.			Qualitative content structuring
Treatment fidelity	Video-assisted observations	IG-Pediatricians	The next preventive medical check-up (U4-U6) of a participating family with psychosocial burden.			Qualitative analysis (incl. Conversation analysis)

Note: t1/ t2/ t3 = first/ second/ third measurement time-point; “U” = U-Untersuchung (preventive medical check-up for children aged 0-3 years)

## Discussion

Representative studies showed that about 13% of the families with small children (aged 0 to 3 years) visiting a pediatrician for a preventive medical check-up have considerable risk factors [7, 5]. Such risk factors increase the probability for psychosocial burden in the family and the vulnerability to severe developmental consequences for the child, making early interventions urgently necessary. In this context, supportive services for burdened families can be beneficial. Although supportive services of the ECI are implemented nationwide, families with psychosocial burden rarely attend these services. The PATH-intervention therefore aims at training the pediatricians’ skills to facilitate the communication with burdened families with small children and the referral to tailored supportive services. Using a prospective quasi-experimental, controlled (matched-pair), longitudinal mixed-method design, we evaluate the target accuracy of this complex healthcare intervention. Quantitative data will allow us to evaluate the extent to which the intervention enables the pediatricians to identify and recommend supportive services to burdened families, and the families’ use of the supportive services of the ECI. Moreover, health-economic analysis will show the cost-effectiveness ratio of the PATH-intervention. Qualitative data will enable us to get a deeper insight into the subjective evaluation and acceptance of the PATH-intervention in the perspective of all the stakeholders involved, as well as in the pediatricians’ treatment fidelity in daily routine care.

By exploring the successful identification of burdened families, recommendation and usage of supportive services, this study could be a starting point for future research. Moreover, the results of this study could form the basis for the broader implementation of the PATH-intervention as standard care.

Nevertheless, some limitations of the study should be mentioned. The evaluation is restricted to two structurally similar German states. Thus, the results may be influenced by specific characteristics of these two states, e.g. their relatively high economic performance. Furthermore, due to an initial briefing session to recall the intervention contents and the selective observations in the IG, as well as due to the study participation itself, it is unavoidable that the pediatricians are sensitized to the issue of families’ psychosocial burden. Therefore, it is possible that the pediatricians will act in a different manner to usual under study participation conditions.

## Conclusion

In summary, due to the great relevance of families’ risk factors and psychosocial burden for both the individual family and the developing child, as well as from a social and economic point of view, this study contributes to an important research field. The results of this study could form a starting point not only for further research, but also for the broader implementation of the PATH-intervention as standard care. The latter could prospectively contribute to the enhancement of the care situation for burdened families with small children.

## Supplementary Information

**Additional file 1: Table S1.** Overview of assessed risk factors, operationalization and calculation of the additive index indicating psychosocial burden.

**Additional file 2.** Family questionnaire about supportive services.

**Additional file 3.** Questionnaire for pediatricians.

**Additional file 4.** Descriptive questionnaire for pediatricians.

**Additional file 5: Table S2.** List of supportive services.

## Data Availability

Not applicable.

## Abbreviations

PATH: Pediatric Attention To Help
ECI: Early Childhood Intervention Program
U1 up to U9: “U-Untersuchung”, medical check-up for infants
NZFH: National Center for Early Prevention
CHWS: Child and youth welfare services
IQCs: Interprofessional Quality Circles
IG: Intervention Group
CG: Controll Group
PHQ: Patient Health Questionnaire
SA: Sensitivity analyses
ICERs: Incremental cost-effectiveness ratios
