# Supplementary material for: Evaluation of a cross-sectoral care intervention for families with psychosocial burden: a study protocol of a controlled trial
Source: BMC Health Serv Res. 2022 Apr 11;22:475. doi: 10.1186/s12913-022-07787-9 (PMC8996544; doi:10.1186/s12913-022-07787-9)
Supplement: Supplementary file 1 — Additional file 1: Table S1. Overview of assessed risk factors, operationalization and calculation of the additive index indicating psychosocial burden. [file 12913_2022_7787_MOESM1_ESM.pdf]

Table S1. *Overview of assessed risk factors, operationalization and calculation of the additive index indicating psychosocial burden.*

Domain	Risk Factor	Description	Number of items	Item content	Scoring	Risk, if score…
a) Family background	Being a single parent	Not living with the biological parent in a partnership in the same household (1)	3	Partnership? Same household? Biological parent?	No = 0; Yes = 1<br>$0 \leq \sum(\text{items}) \leq 3$	= 0
	Low educational qualifications	No professional education and 5 or less years of school education (1)	2	Completed professional education? Highest school degree > 5 years?	No = 0; Yes = 1<br>$0 \leq \sum(\text{items}) \leq 2$	= 0
	Poverty	Receiving social assistance (1)	1	Receiving social assistance in the last 12 months	No = 0; Yes = 1	= 1
	Having many small children	3 or more children aged ≤ 3 years (1)	3	Number of children aged < 1, < 2, < 3	Number of children < 3 years	≥ 3
	Overcrowding	Space per person in household (1–3)	4	Apartment size? Number of persons in household? Number of children < 18 years? Number of children 14 to 17 years?	Apartment size/(1 + 0,7*(Persons in household without oneself) + 0,5*(Children ≥ 14 years))	≤ 24,6 m²
	Conflicting partnership	Frequent quarrels (4)	1	How often vociferous confrontations in the last 6 months?	Never = 1<br>Seldom = 2<br>Sometimes = 3<br>Often = 4<br>Almost always = 5<br>Always = 6	≥ 4
	Lack of social support	No or too little social support for child care (5)	2	Enough people would temporarily take over the care of my child?	Applies completely = 0<br>Rather applies = 1	≥ 5

Domain	Risk Factor	Description	Number of items	Item content	Scoring	Risk, if score…
				Enough people to ask for advice when I am worried about my child?	Rather does not apply = 2<br>Does not apply at all = 3<br>0 ≤ ∑(items) ≤ 6	
(b) Parent's individual preconditions for dealing with care challenges	Young age of the mother	Motherhood at the age of ≤ 21 years (1)	3	Is the respondent the child's biological mother? Age? Which medical check-up for the child?	If the respondent is the biological mother, age of motherhood is calculated by age of mother minus regular time of the medical check-up for the child (e.g. at age 1)	≤ 21
	Doubt about parenting competence	Parenting competence (Parental Stress Index, Subscale Parenting Competence; German version) (6)	4	Education more difficult than expected, uncertain about meeting expectations as a mother, cannot handle tasks, being a parent harder than expected	Applies completely = 5<br>Rather applies = 4<br>Uncertain = 3<br>Rather does not apply = 2<br>Does not apply at all = 1<br>4 ≤ ∑(items) ≤ 20	≥ 11
	Criminal charge of violence	Charged of criminal violence (1)	1	Having been charged of criminal violence	No = 0; Yes = 1	= 1
	Negative childhood experiences	Negative childhood experiences (EMKK) (7)	3	Experienced little love, been treated unfairly, often punished	Applies completely = 3<br>Rather applies = 2<br>Rather does not apply = 1<br>Does not apply at all = 0<br>0 ≤ ∑(items) ≤ 9	≥ 5

Domain	Risk Factor	Description	Number of items	Item content	Scoring	Risk, if score…
(c) Parent's psychological health	Impulsivity	Frequently angry (EMKK) (7)	2	Frequently angry, annoyed by lots of things	Applies completely = 3<br>Rather applies = 2<br>Rather does not apply = 1<br>Does not apply at all = 0<br>$0 \leq \sum(\text{items}) \leq 6$	≥ 4
	Substance abuse	Missed duties because of alcohol or drug use (8)	1	Missed duties because of alcohol or drug use	No = 0; Yes = 1	= 1
	Symptoms of depression and anxiety	Symptoms of depression and anxiety (Patient Health Questionnaire, PHQ-4) (9)	4	Feeling nervous, anxious or on edge, not being able to stop or control worrying, feeling down, depressed or hopeless, little interest or pleasure in doing things	Not at all = 0<br>Several days = 1<br>More than half the days = 2<br>Nearly every day = 3<br>$0 \leq \sum(\text{items}) \leq 12$	≥ 6
(d) Difficulties during pregnancy and in interaction with the child	Unplanned pregnancy	Unplanned pregnancy (1)	1	Pregnancy not planned	No = 0; Yes = 1	= 1
	Prenatal medical care	Regular prenatal check-ups (1)	1	Mother attended prenatal check-ups regularly	No = 0; Yes = 1	= 0
	Negative attribution	Negative attribution of child's affect (EMKK) (7)	1	Impression child cries to provoke parent	No = 0; Yes = 1	= 1
(e) Special care challenges regarding the child	Perinatal complications	Perinatal complications (1)	2	Premature birth, birthweight < 2500g	No = 0; Yes = 1<br>$0 \leq \sum(\text{items}) \leq 2$	≥ 1

Domain	Risk Factor	Description	Number of items	Item content	Scoring	Risk, if score…
	Negative affectivity	Negative affectivity (EMKK) (7)	6	Child not cheerful, child stubborn, has temper tantrums, gets very excited when angry, is ill-tempered, is inconsolable	Applies completely = 4<br>Rather applies = 3<br>Rather does not apply = 2<br>Does not apply at all = 1<br>$6 \leq \sum(\text{items}) \leq 24$	≥ 15
	Disabled or seriously ill child	Disabled or seriously ill child (1)	1	Child disabled or seriously ill	No = 0; Yes = 1	= 1
	Distressing crying behavior	Distressing crying behavior (10)	3	Child cries for more than 3 hours/day, on 3 or more days/week, for 3 or more weeks	No = 0; Yes = 1<br>$0 \leq \sum(\text{items}) \leq 3$	= 3
(f) Problematical caring behavior	Bonding with the child	Lack of parental sensitivity (Parenting Stress Index, Subscale Attachment; German version) (6)	4	Difficulties to empathize with child, hard to find out child's needs, wished to better understand how child feels, takes longer to feel close to child	Applies completely = 5<br>Rather applies = 4<br>Uncertain = 3<br>Rather does not apply = 2<br>Does not apply at all = 1<br>$4 \leq \sum(\text{items}) \leq 20$	≥ 11
	Overreacting	Overreacting (EMKK) (7)	1	Parent treats child sometimes rougher than appropriate	No = 0; Yes = 1	= 1
Overall Score	PSB = Number of risks $[0 \leq \sum(\text{risks}) \leq 23]$<br>PSB ≥ 3 → Psychosocial burden<br>PSB < 3 → No psychosocial burden					

Notes. EMKK = "Erziehungseinstellungen von Müttern mit Kindern im Kleinstkindalter", Educational attitudes of mothers with very children of very young age. The "item content" column reflects the content of the items used. For the original wording, please see the provided references.

References

1. Lorenz S, Ulrich SM, Sann A, Liel C. Self-reported psychosocial stress in parents with small children: Results from the Kinder in Deutschland–KiD-0–3 study. Dtsch Arztebl Int. 2020;117(42):709–16.

2. OECD. 'What are equivalence scales?: OECD project on income distribution and poverty. https://www.oecd.org/els/soc/OECD-Note-EquivalenceScales.pdf. Accessed 16 Feb 2022.

3. Sagner P. Wer wohnt wie groß?: IW-Kurzbericht 11/2021. Köln: Institut der deutschen Wirtschaft; 2021.

4. Schauer M, Ruf-Leuschner M. KINDEX (Interview): Pränatale Erfassung psychosozialer Risiken für eine gesunde Entwicklung – Der Konstanzer INDEX: Universtität Konstanz; 2013. URL: https://www.babyforum-landkreis-konstanz.de/de/aktuelles/KINDEX-Interview.pdf. Accessed 16 Feb 2022.

5. Tröster H. Anforderungen und Belastungen von Müttern mit anfallskranken Kindern. Zeitschrift für Medizinische Psychologie. 1999;8:53–64.

6. Tröster H. Eltern-Belastungs-Inventar (EBI). Deutsche Version des Parenting Stress Index (PSI) von R.R. Abidin. 1st ed. Göttingen: Hogrefe Verlag; 2011.

7. Engfer A. Entwicklung punitiver Mutter-Kind-Interaktionen im sozioökologischen Kontext. Arbeitsbericht zum Antrag an die Deutsche Forschungsgemeinschaft auf Gewährung einer Sachbeihilfe. München: Universität; 1984.

8. Löwe B, Zipfel S, Herzog W. Gesundheitsfragebogen für Patienten (PHQ-D); 2002. https://www.klinikum.uni-heidelberg.de/fileadmin/Psychosomatische_Klinik/pdf_Material/PHQ_Komplett_Fragebogen1.pdf. Accessed 16 Feb 2022.

9. Löwe B, Wahl I, Rose M, Spitzer C, Glaesmer H, Wingenfeld K et al. A 4-item measure of depression and anxiety: validation and standardization of the Patient Health Questionnaire-4 (PHQ-4) in the general population. J Affect Disord. 2010;122(1-2):86–95.

10. Wessel MA, Cobb JC, Jackson EB, Harris, G. S., Jr., Detwiler AC. Paroxysmal fussing in infancy, sometimes called colic. Pediatrics. 1954;14(5):421–35.
