# Supplementary material for: Evaluation of a cross-sectoral care intervention for families with psychosocial burden: a study protocol of a controlled trial
Source: BMC Health Serv Res. 2022 Apr 11;22:475. doi: 10.1186/s12913-022-07787-9 (PMC8996544; doi:10.1186/s12913-022-07787-9)
Supplement: Supplementary file 2 — Additional file 2. Family questionnaire about supportive services. [file 12913_2022_7787_MOESM2_ESM.pdf]

<u>Family questionnaire about supportive services</u>

There are special services to support families with small children. We would like to ask you which of the following supportive services you know, whether your pediatrician informed you about it, as well as whether you have made use of it.

<table>
<tr><td rowspan="5">1.</td><td colspan="3">a) I know about local organizations of the Early Childhood Intervention program.
☐ Yes
☐ No</td></tr>
<tr><td colspan="3">If yes:</td></tr>
<tr><td>b) The pediatrician has informed me about this service.
☐ Yes
☐ No</td><td>If yes:
The information about this service was…
☐ too much
☐ appropriate
☐ too little</td><td>If no::
I would have liked or needed information about this service.
☐ Yes
☐ No</td></tr>
<tr><td colspan="3">c) The pediatrician has specifically recommended me to use this service.
☐ Yes
☐ No</td></tr>
<tr><td colspan="3">d) I have made use of this service.
☐ Yes
☐ No</td></tr>
<tr><td></td><td colspan="3">If you have made use of this service:
How satisfied were you with this service?
☐ Very satisfied
☐ Fairly satisfied
☐ Neither satisfied nor dissatisfied
☐ Fairly dissatisfied
☐ Very dissatisfied</td></tr>
<tr><td></td><td colspan="3">If you have NOT made use of this service:
What were your reasons for not using this service? (You can select multiple answers)
☐ In my local area, the service does not exist.
☐ Due to the Corona virus pandemic, the service is currently not available.
☐ I did not have the time for it.
☐ Our family has no problems, for which it needs such a service.
☐ I do not think this offer will help us.
☐ I am unsure whether I can implement what is suggested to me in this service.
☐ I am worried that someone will try to tell me what to do and what not to do with my child.
☐ I think that I have to manage everything with my child by myself.
☐ I am embarrassed to go to this service.
☐ My family and friends would not approve if I used this service.

I still plan to use this service.
☐ Yes
☐ No</td></tr>
</table>

<table>
<tr><td rowspan="6">2.</td><td colspan="3">a) I know about the onetime welcome visit at home for families with babies.
☐ Yes
☐ No</td></tr>
<tr><td colspan="3">If yes:</td></tr>
<tr><td>b) The pediatrician has informed me about this service.
☐ Yes
☐ No</td><td>If yes:
The information about this service was…
☐ too much
☐ appropriate
☐ too little</td><td>If no::
I would have liked or needed information about this service.
☐ Yes
☐ No</td></tr>
<tr><td colspan="3">c) The pediatrician has specifically recommended me to use this service.
☐ Yes
☐ No</td></tr>
<tr><td colspan="3">d) I have made use of this service.
☐ Yes
☐ No</td></tr>
<tr><td colspan="3"></td></tr>
<tr><td></td><td colspan="3">If you have made use of this service:
How satisfied were you with this service?
☐ Very satisfied
☐ Fairly satisfied
☐ Neither satisfied nor dissatisfied
☐ Fairly dissatisfied
☐ Very dissatisfied</td></tr>
<tr><td></td><td colspan="3">If you have NOT made use of this service:
What were your reasons for not using this service? (You can select multiple answers)
☐ In my local area, the service does not exist.
☐ Due to the Corona virus pandemic, the service is currently not available.
☐ I did not have the time for it.
☐ Our family has no problems, for which it needs such a service.
☐ I do not think this offer will help us.
☐ This service does not fit me and my family.
☐ I am unsure whether I can implement what is suggested to me in this service.
☐ I am worried that someone will try to tell me what to do and what not to do with my child.
☐ I think that I have to manage everything with my child by myself.
☐ I am embarrassed to go to this service.
☐ My family and friends would not approve if I used this service.

I still plan to use this service.
☐ Yes
☐ No</td></tr>
</table>

<table>
<tr><td rowspan="6">3.</td><td colspan="3">a) I know about long-term, periodic care by family midwifes or a pediatric nurse.
☐ Yes
☐ No</td></tr>
<tr><td colspan="3">If yes:</td></tr>
<tr><td>b) The pediatrician has informed me about this service.
☐ Yes
☐ No</td><td>If yes:
The information about this service was…
☐ too much
☐ appropriate
☐ too little</td><td>If no::
I would have liked or needed information about this service.
☐ Yes
☐ No</td></tr>
<tr><td colspan="3">c) The pediatrician has specifically recommended me to use this service.
☐ Yes
☐ No</td></tr>
<tr><td colspan="3">d) I have made use of this service.
☐ Yes
☐ No</td></tr>
<tr><td colspan="3"></td></tr>
<tr><td></td><td colspan="3">If you have made use of this service:
How satisfied were you with this service?
☐ Very satisfied
☐ Fairly satisfied
☐ Neither satisfied nor dissatisfied
☐ Fairly dissatisfied
☐ Very dissatisfied</td></tr>
<tr><td></td><td colspan="3">If you have NOT made use of this service:
What were your reasons for not using this service? (You can select multiple answers)
☐ In my local area, the service does not exist.
☐ Due to the Corona virus pandemic, the service is currently not available.
☐ The family midwife/pediatric nurse was too busy.
☐ I did not have the time for it.
☐ Our family has no problems, for which it needs such a service.
☐ I do not think this offer will help us.
☐ This service does not fit me and my family.
☐ I am unsure whether I can implement what is suggested to me in this service.
☐ I am worried that someone will try to tell me what to do and what not to do with my child.
☐ I think that I have to manage everything with my child by myself.
☐ I am embarrassed to go to this service.
☐ My family and friends would not approve if I used this service.

I still plan to use this service.
☐ Yes
☐ No</td></tr>
</table>

<table>
<tr><td rowspan="6">4.</td><td colspan="3">a) I know about repetitive volunteering visits at home.
☐ Yes
☐ No</td></tr>
<tr><td colspan="3">If yes:</td></tr>
<tr><td>b) The pediatrician has informed me about this service.
☐ Yes
☐ No</td><td>If yes:
The information about this service was…
☐ too much
☐ appropriate
☐ too little</td><td>If no::
I would have liked or needed information about this service.
☐ Yes
☐ No</td></tr>
<tr><td colspan="3">c) The pediatrician has specifically recommended me to use this service.
☐ Yes
☐ No</td></tr>
<tr><td colspan="3">d) I have made use of this service.
☐ Yes
☐ No</td></tr>
<tr><td colspan="3"></td></tr>
<tr><td></td><td colspan="3">If you have made use of this service:
How satisfied were you with this service?
☐ Very satisfied
☐ Fairly satisfied
☐ Neither satisfied nor dissatisfied
☐ Fairly dissatisfied
☐ Very dissatisfied</td></tr>
<tr><td></td><td colspan="3">If you have NOT made use of this service:
What were your reasons for not using this service? (You can select multiple answers)
☐ In my local area, the service does not exist.
☐ Due to the Corona virus pandemic, the service is currently not available.
☐ I did not have the time for it.
☐ Our family has no problems, for which it needs such a service.
☐ I do not think this offer will help us.
☐ This service does not fit me and my family.
☐ I am unsure whether I can implement what is suggested to me in this service.
☐ I am worried that someone will try to tell me what to do and what not to do with my child.
☐ I think that I have to manage everything with my child by myself.
☐ I am embarrassed to go to this service.
☐ My family and friends would not approve if I used this service.

I still plan to use this service.
☐ Yes
☐ No</td></tr>
</table>

<table>
<tr><td rowspan="6">5.</td><td colspan="3">a) I know about counseling services in family or child guidance agencies.
☐ Yes
☐ No</td></tr>
<tr><td colspan="3">If yes:</td></tr>
<tr><td>b) The pediatrician has informed me about this service.
☐ Yes
☐ No</td><td>If yes:
The information about this service was…
☐ too much
☐ appropriate
☐ too little</td><td>If no::
I would have liked or needed information about this service.
☐ Yes
☐ No</td></tr>
<tr><td colspan="3">c) The pediatrician has specifically recommended me to use this service.
☐ Yes
☐ No</td></tr>
<tr><td colspan="3">d) I have made use of this service.
☐ Yes
☐ No</td></tr>
<tr></tr>
<tr><td></td><td colspan="3">If you have made use of this service:
How satisfied were you with this service?
☐ Very satisfied
☐ Fairly satisfied
☐ Neither satisfied nor dissatisfied
☐ Fairly dissatisfied
☐ Very dissatisfied</td></tr>
<tr><td></td><td colspan="3">If you have NOT made use of this service:
What were your reasons for not using this service? (You can select multiple answers)
☐ In my local area, the service does not exist.
☐ Due to the Corona virus pandemic, the service is currently not available.
☐ There were no free slots.
☐ I did not have the time for it.
☐ Our family has no problems, for which it needs such a service.
☐ I do not think this offer will help us.
☐ This service does not fit me and my family.
☐ I am unsure whether I can implement what is suggested to me in this service.
☐ I am worried that someone will try to tell me what to do and what not to do with my child.
☐ I think that I have to manage everything with my child by myself.
☐ I am embarrassed to go to this service.
☐ My family and friends would not approve if I used this service.

I still plan to use this service.
☐ Yes
☐ No</td></tr>
</table>

<table>
<tr><td rowspan="5">6.</td><td colspan="3">a) I know about specific counseling services (e.g. for crying/ sleeping/ feeding the child).
☐ Yes
☐ No</td></tr>
<tr><td colspan="3">If yes:</td></tr>
<tr><td>b) The pediatrician has informed me about this service.
☐ Yes
☐ No</td><td>If yes:
The information about this service was…
☐ too much
☐ appropriate
☐ too little</td><td>If no::
I would have liked or needed information about this service.
☐ Yes
☐ No</td></tr>
<tr><td colspan="3">c) The pediatrician has specifically recommended me to use this service.
☐ Yes
☐ No</td></tr>
<tr><td colspan="3">d) I have made use of this service.
☐ Yes
☐ No</td></tr>
<tr><td></td><td colspan="3">If you have made use of this service:
How satisfied were you with this service?
☐ Very satisfied
☐ Fairly satisfied
☐ Neither satisfied nor dissatisfied
☐ Fairly dissatisfied
☐ Very dissatisfied</td></tr>
<tr><td></td><td colspan="3">If you have NOT made use of this service:
What were your reasons for not using this service? (You can select multiple answers)
☐ In my local area, the service does not exist.
☐ Due to the Corona virus pandemic, the service is currently not available.
☐ There were no free slots.
☐ I did not have the time for it.
☐ Our family has no problems, for which it needs such a service.
☐ I do not think this offer will help us.
☐ This service does not fit me and my family.
☐ I am unsure whether I can implement what is suggested to me in this service.
☐ I am worried that someone will try to tell me what to do and what not to do with my child.
☐ I think that I have to manage everything with my child by myself.
☐ I am embarrassed to go to this service.
☐ My family and friends would not approve if I used this service.

I still plan to use this service.
☐ Yes
☐ No</td></tr>
</table>

<table>
<tr><td rowspan="5">7.</td><td colspan="3">a) I know about services in family or community centers.
☐ Yes
☐ No</td></tr>
<tr><td colspan="3">If yes:</td></tr>
<tr><td>b) The pediatrician has informed me about this service.
☐ Yes
☐ No</td><td>If yes:
The information about this service was…
☐ too much
☐ appropriate
☐ too little</td><td>If no::
I would have liked or needed information about this service.
☐ Yes
☐ No</td></tr>
<tr><td colspan="3">c) The pediatrician has specifically recommended me to use this service.
☐ Yes
☐ No</td></tr>
<tr><td colspan="3">d) I have made use of this service.
☐ Yes
☐ No</td></tr>
<tr><td></td><td colspan="3">If you have made use of this service:
How satisfied were you with this service?
☐ Very satisfied
☐ Fairly satisfied
☐ Neither satisfied nor dissatisfied
☐ Fairly dissatisfied
☐ Very dissatisfied</td></tr>
<tr><td></td><td colspan="3">If you have NOT made use of this service:
What were your reasons for not using this service? (You can select multiple answers)
☐ In my local area, the service does not exist.
☐ Due to the Corona virus pandemic, the service is currently not available.
☐ There were no free slots.
☐ I did not have the time for it.
☐ Our family has no problems, for which it needs such a service.
☐ I do not think this offer will help us.
☐ This service does not fit me and my family.
☐ I am unsure whether I can implement what is suggested to me in this service.
☐ I am worried that someone will try to tell me what to do and what not to do with my child.
☐ I think that I have to manage everything with my child by myself.
☐ I am embarrassed to go to this service.
☐ My family and friends would not approve if I used this service.

I still plan to use this service.
☐ Yes
☐ No</td></tr>
</table>

<table>
<tr><td rowspan="5">8.</td><td colspan="3">a) I know about specific services, e.g. for single parents or immigrants.
☐ Yes
☐ No</td></tr>
<tr><td colspan="3">If yes:</td></tr>
<tr><td>b) The pediatrician has informed me about this service.
☐ Yes
☐ No</td><td>If yes:
The information about this service was…
☐ too much
☐ appropriate
☐ too little</td><td>If no::
I would have liked or needed information about this service.
☐ Yes
☐ No</td></tr>
<tr><td colspan="3">c) The pediatrician has specifically recommended me to use this service.
☐ Yes
☐ No</td></tr>
<tr><td colspan="3">d) I have made use of this service.
☐ Yes
☐ No</td></tr>
<tr><td></td><td colspan="3">If you have made use of this service:
How satisfied were you with this service?
☐ Very satisfied
☐ Fairly satisfied
☐ Neither satisfied nor dissatisfied
☐ Fairly dissatisfied
☐ Very dissatisfied</td></tr>
<tr><td></td><td colspan="3">If you have NOT made use of this service:
What were your reasons for not using this service? (You can select multiple answers)
☐ In my local area, the service does not exist.
☐ Due to the Corona virus pandemic, the service is currently not available.
☐ There were no free slots.
☐ I did not have the time for it.
☐ Our family has no problems, for which it needs such a service.
☐ I do not think this offer will help us.
☐ This service does not fit me and my family.
☐ I am unsure whether I can implement what is suggested to me in this service.
☐ I am worried that someone will try to tell me what to do and what not to do with my child.
☐ I think that I have to manage everything with my child by myself.
☐ I am embarrassed to go to this service.
☐ My family and friends would not approve if I used this service.

I still plan to use this service.
☐ Yes
☐ No</td></tr>
</table>

<table>
<tr><td rowspan="5">9.</td><td colspan="3">a) I know about telephone or Online counseling services.
☐ Yes
☐ No</td></tr>
<tr><td colspan="3">If yes:</td></tr>
<tr><td>b) The pediatrician has informed me about this service.
☐ Yes
☐ No</td><td>If yes:
The information about this service was…
☐ too much
☐ appropriate
☐ too little</td><td>If no::
I would have liked or needed information about this service.
☐ Yes
☐ No</td></tr>
<tr><td colspan="3">c) The pediatrician has specifically recommended me to use this service.
☐ Yes
☐ No</td></tr>
<tr><td colspan="3">d) I have made use of this service.
☐ Yes
☐ No</td></tr>
<tr><td></td><td colspan="3">If you have made use of this service:
How satisfied were you with this service?
☐ Very satisfied
☐ Fairly satisfied
☐ Neither satisfied nor dissatisfied
☐ Fairly dissatisfied
☐ Very dissatisfied</td></tr>
<tr><td></td><td colspan="3">If you have NOT made use of this service:
What were your reasons for not using this service? (You can select multiple answers)
☐ I did not have the time for it.
☐ Our family has no problems, for which it needs such a service.
☐ I do not think this offer will help us.
☐ This service does not fit me and my family.
☐ I am unsure whether I can implement what is suggested to me in this service.
☐ I am worried that someone will try to tell me what to do and what not to do with my child.
☐ I think that I have to manage everything with my child by myself.
☐ I am embarrassed to go to this service.
☐ My family and friends would not approve if I used this service.

I still plan to use this service.
☐ Yes
☐ No</td></tr>
</table>

<table>
<tr><td rowspan="5">10.</td><td colspan="3">a) I know about trainings for parents.
☐ Yes
☐ No</td></tr>
<tr><td colspan="3">If yes:</td></tr>
<tr><td>b) The pediatrician has informed me about this service.
☐ Yes
☐ No</td><td>If yes:
The information about this service was…
☐ too much
☐ appropriate
☐ too little</td><td>If no::
I would have liked or needed information about this service.
☐ Yes
☐ No</td></tr>
<tr><td colspan="3">c) The pediatrician has specifically recommended me to use this service.
☐ Yes
☐ No</td></tr>
<tr><td colspan="3">d) I have made use of this service.
☐ Yes
☐ No</td></tr>
<tr><td></td><td colspan="3">If you have made use of this service:
How satisfied were you with this service?
☐ Very satisfied
☐ Fairly satisfied
☐ Neither satisfied nor dissatisfied
☐ Fairly dissatisfied
☐ Very dissatisfied</td></tr>
<tr><td></td><td colspan="3">If you have NOT made use of this service:
What were your reasons for not using this service? (You can select multiple answers)
☐ In my local area, the service does not exist.
☐ Due to the Corona virus pandemic, the service is currently not available.
☐ There were no free slots.
☐ I did not have the time for it.
☐ Our family has no problems, for which it needs such a service.
☐ I do not think this offer will help us.
☐ This service does not fit me and my family.
☐ I am unsure whether I can implement what is suggested to me in this service.
☐ I am worried that someone will try to tell me what to do and what not to do with my child.
☐ I think that I have to manage everything with my child by myself.
☐ I am embarrassed to go to this service.
☐ My family and friends would not approve if I used this service.

I still plan to use this service.
☐ Yes
☐ No</td></tr>
</table>

<table>
<tr><td rowspan="5">11.</td><td colspan="3">a) I know about parent-child-groups.
☐ Yes
☐ No</td></tr>
<tr><td colspan="3">If yes:</td></tr>
<tr><td>b) The pediatrician has informed me about this service.
☐ Yes
☐ No</td><td>If yes:
The information about this service was…
☐ too much
☐ appropriate
☐ too little</td><td>If no::
I would have liked or needed information about this service.
☐ Yes
☐ No</td></tr>
<tr><td colspan="3">c) The pediatrician has specifically recommended me to use this service.
☐ Yes
☐ No</td></tr>
<tr><td colspan="3">d) I have made use of this service.
☐ Yes
☐ No</td></tr>
<tr><td></td><td colspan="3">If you have made use of this service:
How satisfied were you with this service?
☐ Very satisfied
☐ Fairly satisfied
☐ Neither satisfied nor dissatisfied
☐ Fairly dissatisfied
☐ Very dissatisfied</td></tr>
<tr><td></td><td colspan="3">If you have NOT made use of this service:
What were your reasons for not using this service? (You can select multiple answers)
☐ In my local area, the service does not exist.
☐ Due to the Corona virus pandemic, the service is currently not available.
☐ There were no free slots.
☐ I did not have the time for it.
☐ Our family has no problems, for which it needs such a service.
☐ I do not think this offer will help us.
☐ This service does not fit me and my family.
☐ I am unsure whether I can implement what is suggested to me in this service.
☐ I am worried that someone will try to tell me what to do and what not to do with my child.
☐ I think that I have to manage everything with my child by myself.
☐ I am embarrassed to go to this service.
☐ My family and friends would not approve if I used this service.

I still plan to use this service.
☐ Yes
☐ No</td></tr>
</table>

<table>
<tr><td rowspan="6">12.</td><td colspan="3">a) I know about services concerning early fostering (e.g. developmental retardation).
☐ Yes
☐ No</td></tr>
<tr><td colspan="3">If yes:</td></tr>
<tr><td>b) The pediatrician has informed me about this service.
☐ Yes
☐ No</td><td>If yes:
The information about this service was…
☐ too much
☐ appropriate
☐ too little</td><td>If no::
I would have liked or needed information about this service.
☐ Yes
☐ No</td></tr>
<tr><td colspan="3">c) The pediatrician has specifically recommended me to use this service.
☐ Yes
☐ No</td></tr>
<tr><td colspan="3">d) I have made use of this service.
☐ Yes
☐ No</td></tr>
<tr><td colspan="3"></td></tr>
<tr><td></td><td colspan="3">If you have made use of this service:
How satisfied were you with this service?
☐ Very satisfied
☐ Fairly satisfied
☐ Neither satisfied nor dissatisfied
☐ Fairly dissatisfied
☐ Very dissatisfied</td></tr>
<tr><td></td><td colspan="3">If you have NOT made use of this service:
What were your reasons for not using this service? (You can select multiple answers)
☐ In my local area, the service does not exist.
☐ Due to the Corona virus pandemic, the service is currently not available.
☐ There were no free slots.
☐ I did not have the time for it.
☐ Our family has no problems, for which it needs such a service.
☐ I do not think this offer will help us.
☐ This service does not fit me and my family.
☐ I am unsure whether I can implement what is suggested to me in this service.
☐ I am worried that someone will try to tell me what to do and what not to do with my child.
☐ I think that I have to manage everything with my child by myself.
☐ I am embarrassed to go to this service.
☐ My family and friends would not approve if I used this service.

I still plan to use this service.
☐ Yes
☐ No</td></tr>
</table>

13.	a) I know about **support by midwifes after the child's birth**.<br>☐ Yes<br>☐ No
	If yes:
	b) I have made use of this service.<br>☐ Yes<br>☐ No

14.	a) I know about **medical services for mothers after the child's birth**.<br>☐ Yes<br>☐ No
	If yes:
	b) I have made use of this service.<br>☐ Yes<br>☐ No
