# Supplementary material for: Evaluation of a cross-sectoral care intervention for families with psychosocial burden: a study protocol of a controlled trial
Source: BMC Health Serv Res. 2022 Apr 11;22:475. doi: 10.1186/s12913-022-07787-9 (PMC8996544; doi:10.1186/s12913-022-07787-9)
Supplement: Supplementary file 3 — Additional file 3. Questionnaire for pediatricians. [file 12913_2022_7787_MOESM3_ESM.pdf]

Nationales Zentrum Frühe Hilfen

UNIVERSITÄTS KLINIKUM FREIBURG

Sektion Versorgungsforschung und Rehabilitationsforschung (SEVERA)

Gefördert durch:

Gemeinsamer Bundesausschuss Innovationsausschuss

Questionnaire for Pediatricians

- Assessment of the family's psychosocial burden -

Intervention Group

Please answer the following questions after completing the preventive medical check-up with a participating family.

Thank you very much!

1. Do you consider the family to be psychosocially burdened?

- ❑ No ⇨ **Please continue with question 6!**
- ❑ Yes

⇩

2. How severe do you consider the psychosocial burden of the family?

- ❑ low burden
- ❑ medium burden
- ❑ severe burden
- ❑ extremely severe burden

3. How would you describe the family according to the one-day training program on „Identifying psychosocially burdened families and motivational interviewing”?

- ❑ „green family“ (the family seeks advice and is informed)
- ❑ „yellow family“ (the family seeks advice but is not informed)
- ❑ „red family“ (the family neither seeks advice nor is informed)

4. Have you addressed the perceived psychosocial burden of the family?

- ❑ Yes ⇨ **Please continue with question 5!**
- ❑ No ⇨ **Please continue with question 6!**

5. How well did you succeed in addressing the perceived psychosocial burden with the parents during the preventive medical check-up?

- ❑ very good
- ❑ quite good
- ❑ medium
- ❑ quite bad
- ❑ very bad

6. How do you consider the need for supportive services for this family?

- ❑ no need
- ❑ low need
- ❑ medium need
- ❑ great need

Nationales Zentrum Frühe Hilfen

UNIVERSITÄTS KLINIKUM FREIBURG

Sektion Versorgungsforschung und Rehabilitationsforschung (SEVERA)

Gefördert durch:

Gemeinsamer Bundesausschuss Innovationsausschuss

Questionnaire for Pediatricians

- Assessment of the family's psychosocial burden -

Control Group

Please answer the following questions after completing the preventive medical check-up with a participating family.

Thank you very much!

1. Do you consider the family to be psychosocially burdened?

- ❑ No ⇨ **Please continue with question 5!**
- ❑ Yes

⇩

2. How severe do you consider the psychosocial burden of the family?

- ❑ low burden
- ❑ medium burden
- ❑ severe burden
- ❑ extremely severe burden

3. Have you addressed the perceived psychosocial burden of the family?

- ❑ Yes ⇨ **Please continue with question 4!**
- ❑ No ⇨ **Please continue with question 5!**

4. How well did you succeed in addressing the perceived psychosocial burden with the parents during the preventive medical check-up?

- ❑ very good
- ❑ quite good
- ❑ medium
- ❑ quite bad
- ❑ very bad

5. How do you consider the need for supportive services for this family?

- ❑ no need
- ❑ low need
- ❑ medium need
- ❑ great need
