# Supplementary material for: Evaluation of a cross-sectoral care intervention for families with psychosocial burden: a study protocol of a controlled trial
Source: BMC Health Serv Res. 2022 Apr 11;22:475. doi: 10.1186/s12913-022-07787-9 (PMC8996544; doi:10.1186/s12913-022-07787-9)
Supplement: Supplementary file 4 — Additional file 4. Descriptive questionnaire for pediatricians. [file 12913_2022_7787_MOESM4_ESM.pdf]

Gefördert durch:

Sektion Versorgungsforschung und Rehabilitationsforschung (SEVERA)

Descriptive Questionnaire for Pediatricians

In the following, we would like to ask you some questions about yourself and your practical experiences as well as about some structural characteristics of your office.

Please read each question carefully and answer all questions. Please select only one answer option. There are no right or wrong answers. Please answer each question as it fits to you and your practice. It will take about 5 minutes to complete this questionnaire.

Thank you very much!

Questions about yourself and your office:

1. **How old are you?**

   _______________ years

2. **Your gender?**

   ❑ woman ❑ man ❑ divers

3. **How long have you been working as a pediatrician?**

   For ____________________ year(s)

4. **How long have you been working as a pediatrician in a private practice?**

   For ____________________ year(s)

5. **In which professional context do you work?**

   I am a …

   ❑ registered doctor in a private practice.

   ❑ registered doctor in a private practice joint with other pediatricians.

   If yes: How many doctors are working in the joint private practice?

   _______________ doctors

   ❑ employee in a private practice.

The following questions address characteristics of the families visiting your office and aspects of your pediatric work practice.

6. **How do you consider the living conditions of the families who visit your office?**

   Families visiting my office come …

   ❑ … predominantly from difficult socio-economic backgrounds

   ❑ … in about equal proportions from difficult and stable socio-economic backgrounds

   ❑ … predominantly from stable socio-economic backgrounds

7. **How many preventive check-ups do you approximately carry out per month?**

   about _____________ preventive check-ups

8. **With regard to families visiting your office for the preventive medical check-ups „U3-U7a“:**
   How large do you estimate the proportion of families with psychosocial burdens that are relevant for the child’s development?

   about _____________% of the families

9. **How large do you estimate the proportion of psychosocially burdened families, for whom supportive services would be helpful?**

   about _____________% of the psychosocially burdened families

10. **How confident do you feel in identifying families with psychosocial burden?**

    ❑ very unconfident

    ❑ unconfident

    ❑ quite unconfident

    ❑ neither unconfident nor confident

    ❑ quite confident

    ❑ confident

    ❑ very confident

11. Do you use a questionnaire to assess psychosocial burden in families?

❑ Yes

If yes: Which questionnaire do you use? ____________________________

❑ No

12. How confident do you feel in motivating psychosocially burdened families to use supportive offers?

❑ very unconfident

❑ unconfident

❑ quite unconfident

❑ neither unconfident nor confident

❑ quite confident

❑ confident

❑ very confident

13. Have you already participated in a training program concerning the Early Childhood Intervention (ECI) program/ supportive services for families with psychosocial burden?

❑ Yes, namely: ____________________________________

❑ No

14. How familiar are you with the concept of the Early Childhood Intervention (ECI) program?

□ not at all familiar

□ a little familiar

□ more or less familiar

□ quite familiar

□ absolutely familiar

15. To what extent is your office networked with supportive services of the Early Childhood Intervention (ECI) program?

- ❑ not at all
- ❑ a little
- ❑ to some extent
- ❑ strongly
- ❑ very strong

16. Do you regularly take part in (inter-)professional exchanges (e.g. regular meetings, quality circles) where networking with the child and youth welfare services is addressed?

- ❑ Yes, namely: ____________________________________
- ❑ No

17. Do your office have special services for psychosocially burdened families (e.g. socio-pedagogical counselling hours)?

- ❑ Yes, namely: ____________________________________
- ❑ No

18. Did your office participate in other scientific studies while participating in this study?

- ❑ Yes, namely: ____________________________________
- ❑ No
