# Supplementary material for: Evaluation of a cross-sectoral care intervention for families with psychosocial burden: a study protocol of a controlled trial
Source: BMC Health Serv Res. 2022 Apr 11;22:475. doi: 10.1186/s12913-022-07787-9 (PMC8996544; doi:10.1186/s12913-022-07787-9)
Supplement: Supplementary file 5 — Additional file 5: Table S2. List of supportive services. [file 12913_2022_7787_MOESM5_ESM.pdf]

Table S2. *List of supportive services*

No.	Supportive services
1	Local organizations of the Early Childhood Intervention program
2	Onetime welcome visit at home for families with babies
3	Long-term, periodic care by family midwifes or a pediatric nurse
4	Repetitive volunteering visits at home
5	Counseling services in family or child guidance agencies
6	Specific counseling services (e.g. for crying/ sleeping/ feeding the child)
7	Services in family or community centers
8	Specific services, e.g. for single parents or immigrants
9	Telephone or Online counseling services
10	Trainings for parents
11	Parent-child-groups
12	Services concerning early fostering (e.g. developmental retardation)
13	Support by midwifes after the child’s birth
14	Medical services for mothers after the child’s birth
